# Supplementary material for: Should Neurosurgeons Try to Preserve Non-Traditional Brain Networks? A Systematic Review of the Neuroscientific Evidence
Source: J Pers Med. 2022 Apr 6;12(4):587. doi: 10.3390/jpm12040587 (PMC9029431; doi:10.3390/jpm12040587)
Supplement: Supplementary file 1 [file jpm-12-00587-s001.zip › jpm-1628442-supplementary.pdf]

Should Neurosurgeons Try to Preserve Non-Traditional Brain Networks?: A Systematic Review of the Neuroscientific Evidence

Abbreviations List

CEN, central executive network
DAN, dorsal attention network
DMN, default mode network
VAN, ventral attention network
SN, salience network
SMA, supplementary motor area

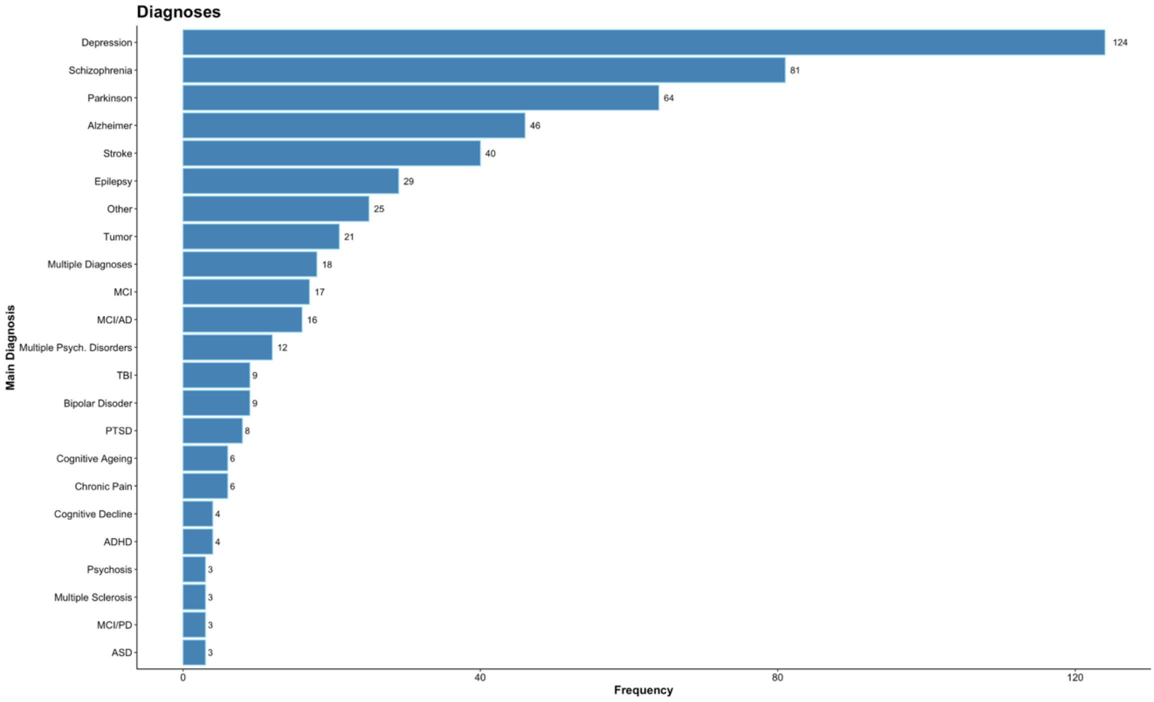

Figure S1. Frequency of diagnoses in all included studies.

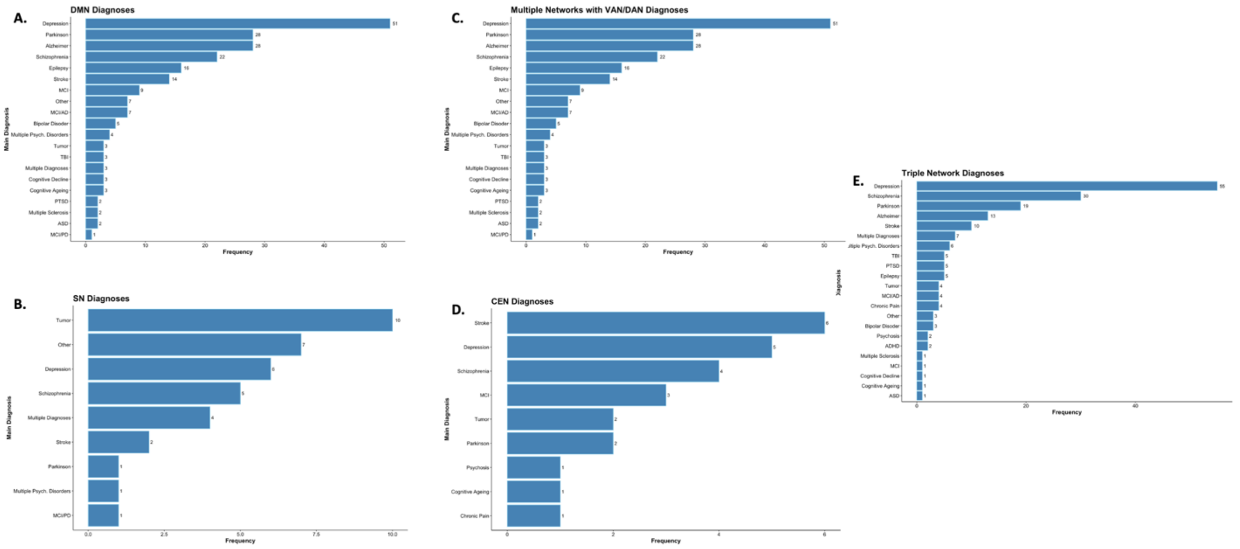

Figure S2. Frequency of diagnoses in all included studies according to network affiliation. Diagnoses are demonstrated according to the (A) default mode network (DMN), (B) salience network (SN), (C) central executive network (CEN), (D) ventral attention network (VAN) or dorsal attention network (DAN) in combination with each other or the other networks, and (E) triple network combination of DMN, SN, and CEN.

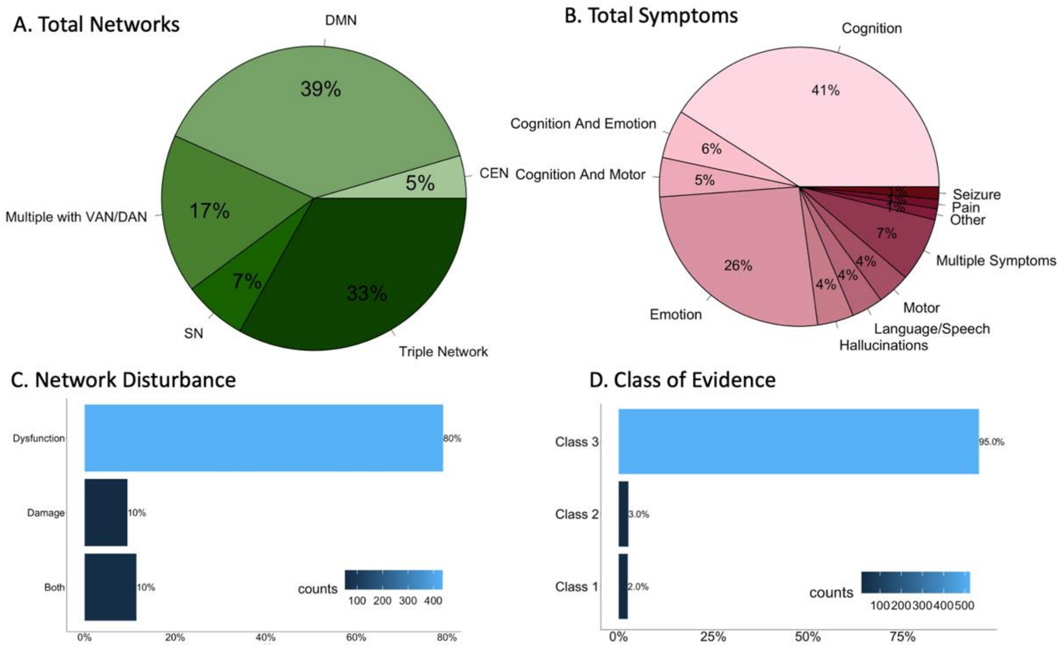

Figure S3. Breakdown of Included Studies. (A) The most common networks identified in isolation or in the most common combinations are demonstrated based on their relative proportion of all included studies. Combinations of networks were categorized into cognitive control networks (DMN, CEN, SN) or any other network in combination with the attention networks (VAN/DAN). Only one study was involving the DAN alone and therefore was excluded from the figure 3a. Precents add up to above 101% due to differences in rounding. (B) Deficits associated with network disfunction according to their relative proportion based on network affiliation. (C) Damage or dysfunction in a network was decided based on if a study examined the functional or structural

connectivity of a network, respectively. (D) The relative proportion of studies per class of evidence is demonstrated.
